# Bacteriophage-Based Bacterial Wilt Biocontrol for an Environmentally Sustainable Agriculture

**DOI:** 10.3389/fpls.2017.01218

**Published:** 2017-07-14

**Authors:** Belén Álvarez, Elena G. Biosca

**Affiliations:** ^1^Departamento de Investigación Aplicada y Extensión Agraria, Instituto Madrileño de Investigación y Desarrollo Rural, Agrario y Alimentario Madrid, Spain; ^2^Departamento de Microbiología y Ecología, Universitat de València Valencia, Spain

**Keywords:** biological agent, lytic phage, lysogenic phage, treatment, management

## Abstract

Bacterial wilt diseases caused by *Ralstonia solanacearum*, *R. pseudosolanacearum*, and *R. syzygii* subsp. *indonesiensis* (former *R. solanacearum* species complex) are among the most important plant diseases worldwide, severely affecting a high number of crops and ornamentals. Difficulties of bacterial wilt control by non-biological methods are related to effectiveness, bacterial resistance and environmental impact. Alternatively, a great many biocontrol strategies have been carried out, with the advantage of being environmentally friendly. Advances in bacterial wilt biocontrol include an increasing interest in bacteriophage-based treatments as a promising re-emerging strategy. Bacteriophages against the bacterial wilt pathogens have been described with either lytic or lysogenic effect but, they were proved to be active against strains belonging to *R. pseudosolanacearum* and/or *R. syzygii* subsp. *indonesiensis*, not to the present *R. solanacearum* species, and only two of them demonstrated successful biocontrol potential *in planta*. Despite the publication of three patents on the topic, until now no bacteriophage-based product is commercially available. Therefore, there is still much to be done to incorporate valid bacteriophages in an integrated management program to effectively fight bacterial wilt in the field.

## Introduction

**FIGURE 1:**
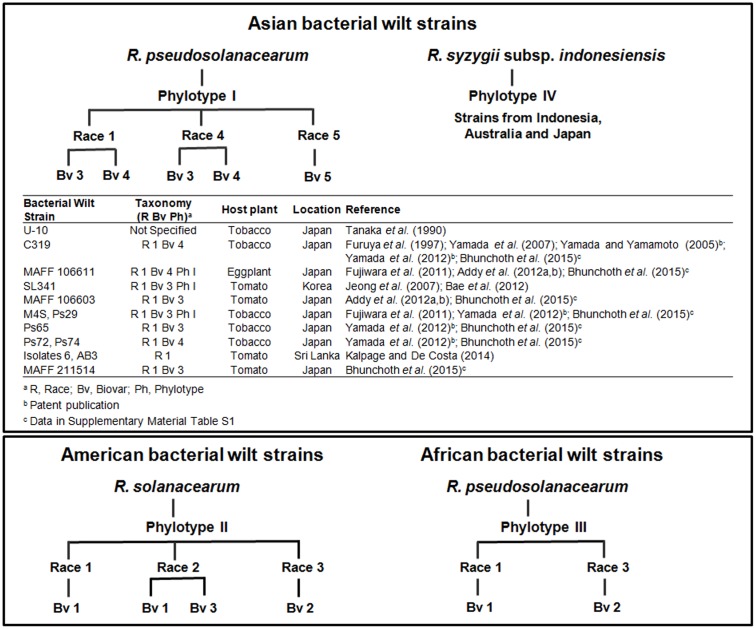
Bacterial wilt strains used to test the biocontrol potential of the bacteriophages *in planta*. Correspondence among the geographical origin of bacterial wilt strains, the species *Ralstonia pseudosolanacearum*, *R. syzygii* subsp. *indonesiensis*, and present *R. solanacearum* (Safni et al., 2014) (former “*R. solanacearum* species complex” according to Fegan and Prior, 2005), and previous infraspecific classifications of the species complex in four phylotypes, five races, and five biovars, according to molecular characteristics (Fegan and Prior, 2005), host range (Buddenhagen and Kelman, 1964; Hayward, 1991) and biochemical properties (Hayward, 1964, 1991), respectively. To date, all bacteriophage-based biocontrol assays were performed against Asian bacterial wilt strains, presently not classified as *R. solanacearum*.

The species *Ralstonia solanacearum*, *R.* *pseudosolanacearum*, and *R. syzygii* subsp. *indonesiensis* (Safni et al., 2014) are the causative agents of bacterial wilt (Kelman, 1953; Hayward, 1991), a disease with a worldwide distribution (Elphinstone, 2005; EPPO, 2017). During the last decade, they have been considered the “*R. solanacearum* species complex” (Fegan and Prior, 2005), still known as *R. solanacearum*, the name that will be maintained in this review. *R. solanacearum* has been traditionally classified in five biovars, five races, and four phylotypes, according to biochemical properties (Hayward, 1964, 1991), host range (Buddenhagen and Kelman, 1964; Hayward, 1991) and molecular characteristics (Fegan and Prior, 2005), respectively (**Figure 1**). Currently, this pathogen affects more than 400 plant species, including strategic solanaceous crops and ornamental plants. The effects are particularly harmful on potato or tomato because they are staple crops. Harvest losses can reach up to 100% in banana, 90% in tomato and potato, 30% in tobacco and 20% in peanut (Elphinstone, 2005). Symptoms caused by this plant pathogenic bacterium are progressive wilting of the plant and rotting of potato tubers. *R. solanacearum* has been ranked in the second place in the top 10 list of more devastating plant pathogenic bacteria (Mansfield et al., 2012). This pathogen is provided with a wide range of virulence and pathogenicity factors (Schell, 2000; Genin and Denny, 2012; Peeters et al., 2013), a high number of effectors (Poueymiro and Genin, 2009; Deslandes and Genin, 2014) and novel virulence-associated functions (Genin and Denny, 2012; Poueymiro et al., 2014). The bacterium infects the host colonizing the xylem (Vasse et al., 1995), reaching high populations which activate a *quorum sensing* system with PhcA as a key transcriptional regulator (Schell, 2000), and eventually causing plant wilting. The pathogen can then return to the environment, where it disseminates mainly through plant material, soil, weeds, and water (Elphinstone et al., 1998; Elphinstone, 2005; López and Biosca, 2005; Álvarez et al., 2010), and can persist in these reservoirs under adverse conditions for long periods by different strategies, retaining pathogenicity (Álvarez et al., 2008, 2010).

The numerous pathogenicity determinants, wide host range and ability to survive of *R. solanacearum* make it difficult the control of the bacterial wilt disease, particularly by chemicals and/or physical treatments, as well as cultural practices. The application of copper compounds and other agrochemicals has a strong environmental impact, and is often related to the appearance of bacterial resistance or even the viable but non-culturable state (Grey and Steck, 2001; Yuliar et al., 2015). Alternatively, numerous biocontrol strategies have been carried out, which also have the advantage of being environmentally friendly. Bacterial wilt biocontrol has been mainly focused on the use of antagonistic microorganisms, usually avirulent mutants of the pathogen and strains of other bacterial species, and also some fungi and bacteriophages (Yuliar et al., 2015; Buttimer et al., 2017).

Advances in biocontrol measures for bacterial wilt include an increasing interest in bacteriophages (viruses specifically infecting bacteria), which are the most abundant microorganisms regulating bacterial populations in the environment. Many of them have a host range restricted to one or few related bacterial species, and consequently, are potential biocontrol agents capable of targeting bacterial pathogens without environmental risk on either the crop to be protected or the surrounding environmental microbiota. According to their life cycle they can be lytic or lysogenic. Lytic phages proliferate and destroy the host bacterial cell, being more effective against the target bacteria, whereas lysogenic phages integrate their genome into the genome of the bacterial host and replicate without destroying the bacterial cell. Thus, bacterial infectious diseases can be treated by the action of phages, a biocontrol method also known as phage therapy. This method can be considered as a promising strategy against bacterial wilt disease (Fujiwara et al., 2011; Bae et al., 2012; Bhunchoth et al., 2015), since it has been successfully applied for controlling some important plant diseases caused by phytopathogenic bacteria (Jones et al., 2007; Balogh et al., 2010; Doffkay et al., 2015; Buttimer et al., 2017).

## Bacteriophage-Based Bacterial Wilt Biocontrol

Biocontrol of *R. solanacearum* has been described with either lytic or lysogenic bacteriophages and has been carried out in different ways with variable results (**Table 1**). Although in most of these cases the isolated phages were active against strains presently not classified as *R. solanacearum* (**Figure 1**), this nomenclature will be maintained in this review, according to the published works.

**Table 1:** *Ralstonia solanacearum* (Rsol) bacteriophages used for bacterial wilt (BW) biocontrol *in planta* and detail of the experimental procedures.

Bacteriophage					Biocontrol assay experimental conditions				
	
Code	Taxonomy	Location	Source	Infection cycle	Rsol host	Target crop	Bacteriophage inoculation procedure	Pots / Assay	BW reduction	Reference
P4282	*Myoviridae* (Yamada et al., 2007)	Japan	Stems of wilted tobacco	Lytic	U-10	Tobacco	Preinoculation with avirulent Rsol strain M4S (10^8^ cfu/ml) + watering with P4282 (10^7^ pfu/ml) 4 days before inoculation of injured roots with virulent strain U-10 (10^7^ cfu/ml)	20	>75%	Tanaka et al., 1990

ϕRSS1	*Inoviridae*	Japan	Soil	Lysogenic	C319	Tobacco	Injection of 1 μl of ϕRSS1-infected C319 cells (10^7^ cells) into the stem of tobacco plants	5	0%	Yamada et al., 2007

ϕRSL1	*Myoviridae*	Japan	Soil	Lytic	MAFF 106611	Tomato	Soaking with a phage or phage mixture (1.3 × 10^10^ pfu/pot) twice in 1-month interval, 2 days later inoculation with MAFF 106611 (10^8^ cells/ml) by 30 s dipping in injured roots	11	100%	Fujiwara et al., 2011
									
ϕRSA1 +	(ϕRSA1,							11	0%	
ϕRSB1	ϕRSL1)									
									
ϕRSA1 +	*Podoviridae*							11	0%	
ϕRSB1 +	(ϕRSB1)									
ϕRSL1										

PE204	*Podoviridae*	Korea	Pepper field (Murugaiyan et al., 2010)	Lytic	SL341	Tomato	Drenching of soil and roots simultaneously with 2 ml of PE204 (10^8^ pfu/ml) + SL341 (10^7^ cfu/g soil)	8–10	100%	Bae et al., 2012
								
							Drenching of soil and roots with 2 ml of PE204 (10^8^ pfu/ml) prior to inoculation with SL341 (10^7^ cfu/g soil)	8–10	0%	
								
							Drenching of soil and roots with strain SL341 (10^7^ cfu/g soil), and inoculation with PE204 (10^8^ pfu/ml)	8–10	30–60%	

φRSS1	*Inoviridae*	Japan	Soil (Yamada et al., 2007)	Lysogenic	MAFF 106603, 106611 (not shown)	Tomato	Injection of 3 μl of φRSS1-infected MAFF 106603 (10^5^ cells) into the stem of tomato plants	10	0%	Addy et al., 2012a

φRSM3	*Inoviridae*	Japan	(Askora et al., 2009)	Lysogenic			Injection of 1 μl of φRSM3-infected MAFF 106603 (10^5^ cells) into the stem of tomato plants	20	100%	Addy et al., 2012b

C + F + J + N + O + P	Not Specified	Sri Lanka	Soil from fields or rich with organic matter	Lytic	Isolate 6	Tomato	Drenching of soil and roots with phage mixture (2.86 × 10^6^ pfu/ml) twice before and once after the	10	10%	Kalpage and De Costa, 2014
									
					Isolate AB3		inoculation with isolate (1 × 10^8^ cfu/ml) within a 2 day-interval or on the 14th day after transplanting and then inoculation with the isolate	10	20%	

J2	*Podoviridae*	Thailand	Soil from tomato field	Lytic	MAFF 211514	Tomato	Drenching of soil and roots with 5 ml of J2 (2 × 10^10^ pfu/ml) 1 day before inoculation of injured roots with MAFF 211514 (1 × 10^6^ cfu/g soil)	4	50%	Bhunchoth et al., 2015


Okabe and Goto (1963) reviewed the bacteriophages in relation to plant diseases, with a number of papers concerning the phages of *R. solanacearum*. Thus, a lytic phage named S1 active against three out of 40 strains of the pathogen, and five lysogenic phages affecting different strains, were reported. Attempts to use phages for bacterial wilt control were made, some of them with success. These assays were performed by inoculations with a mixture of the phage and the bacterium, or by previous treatment of plants or seeds with the phage (Okabe and Goto, 1963). The same authors reported that, when tomato or tobacco plants were set in a soil infested with the phage-bacterium mixture without successful biocontrol, the phage could be reisolated from the wilted stems (Okabe and Goto, 1963). As a part of an integrated biocontrol treatment, plant inoculations with bacteriophage P4282, isolated from wilted tobacco stems in Japan, were performed (Tanaka et al., 1990). P4282 was added with and without an avirulent strain of the pathogen named M4S. Subsequently, inoculations of the same plants with a virulent strain of the pathogen revealed that the combined treatment of the phage P4282 plus the avirulent bacterial strain M4S was more effective in reducing the incidence and severity of tobacco wilt (**Table 1**) than the use of the avirulent strain alone (Tanaka et al., 1990).

In addition to phage P4282, there were other lytic bacteriophages with similar infective ability but, with limited host range (Tanaka et al., 1990; Toyoda et al., 1991*;* Ozawa et al., 2001), and so biocontrol assays *in planta* were not carried out with them. Yamada et al. (2007) described the isolation of four types of bacteriophages (φRSL, φRSA, φRSM, and φRSS) that specifically infected strains from soil samples taken in different areas of Japan. These authors performed a morphological and molecular characterization, and some lytic tests with bacterial cultures. Two of them (φRSA1 and φRSL1) were *Myovirus*-type bacteriophages, with double-stranded (ds) DNA genomes of very different size (39 and 240 kb, respectively), and phage φRSA1 having ability to establish lysogenic cycle. The other two (φRSM1 and φRSS1) were *Inovirus*-type filamentous bacteriophages (Ff-type). Biocontrol assays were performed by injecting one of the lysogenic bacteriophages (φRSS1) into the major stem of the tested plants, and it was concluded that this filamentous bacteriophage was not successful for bacterial wilt biocontrol (Yamada et al., 2007) (**Table 1**). It was then suggested the utility of bacteriophages φRSM1 and φRSS1 for molecular biological studies and specific and efficient detection of *R. solanacearum*, as well as the convenience of phages φRSL1 and φRSA1 for biocontrol assays of the pathogen in plant crops (Yamada et al., 2007). Another bacteriophage with lytic activity against *R. solanacearum* was isolated in Japan, named φRSB1 (Kawasaki et al., 2009) and classified into the *Podoviridae*-like family, with dsDNA of approximately 43.0 kb. The host range of phage φRSB1 included 13 out of 15 *R. solanacearum* strains, involving races 1, 3, and 4, and biovars 3, 4, and N2 and, therefore, most of them not belonging to the present *R. solanacearum* species (**Figure 1**). All of these three bacteriophages (φRSA1, φRSB1, and φRSL1) were later proposed for biocontrol potential *in planta* (Fujiwara et al., 2011) due to their *in vitro* lytic activity against a relatively large host range of strains. However, although the activity of bacteriophages φRSA1 and φRSB1 (tested separately or in combination) and/or with φRSL1 was effective, soon afterwards populations of resistant *R. solanacearum* cells appeared (Fujiwara et al., 2011). Therefore, *in planta* biocontrol assays with φRSB1 or φRSA1 were discarded, and only mixtures of them and/or φRSL1, or φRSL1 alone, were considered as biocontrol treatments. The best results were achieved with phage φRSL1, revealing a good potential for bacterial wilt biocontrol (**Table 1**). Nevertheless, although phage pretreatment of tomato seedlings reduced root colonization by the pathogen and no disease symptoms were observed, there was a remaining coexistence of the bacteriophage and the pathogen (Fujiwara et al., 2011). The phage φRSL1 is considered a large-tailed (jumbo) bacteriophage defining a new lineage of the *Myoviridae* family, and was isolated from crop fields (Yamada et al., 2007, 2010). The genome size of φRSL1 is 231,255 kb, with a total of 343 *orfs* grouped into four genomic regions. Phage particles consist of an icosahedral head of 150 nm in diameter and a long contractile tail that is 138 nm long and 22.5 nm wide (Yamada et al., 2010). φRSL1 lytic activity *in vitro* results in clear plaques with 17 out of 18 bacterial wilt strains of races 1 and 3, and biovars 3 and 4 (Yamada et al., 2010).

A second lytic bacteriophage with a good potential for bacterial wilt biocontrol was PE204, of the *Podoviridae* family, isolated from a pepper field in Korea (Murugaiyan et al., 2010; Bae et al., 2012). PE204 completely inhibited the incidence of bacterial wilt after simultaneous application with the pathogen in the rhizosphere of tomato plants. It was also found that, while pretreatment with the phage was ineffective, posttreatment delayed disease development (Bae et al., 2012) (**Table 1**). Morphological and genomic analysis of PE204 phage particles revealed that it was almost identical to φRSB1 (Kawasaki et al., 2009), a T7-like phage. PE204 particles had a head of about 60 nm in diameter and a short tail of 16–20 nm in length. The total PE204 DNA sequence was 21 kb, and included the *orf1-4*, *orf9*, *orf17*, *orf22-32*, *orf37*, and *orf38* of φRSB1, with a partial genome organization identical to this phage (Bae et al., 2012).

The filamentous bacteriophage φRSS1 (Yamada et al., 2007) was anew assayed to verify the efficacy of bacterial wilt biocontrol (Addy et al., 2012a) (**Table 1**). In this case, when cells of the pathogen were infected with φRSS1 and injected into tomato plants, an increase in the bacterial virulence was observed, since the infection induced an early expression of the key regulatory *phcA* gene, interfering the main *R. solanacearum quorum sensing* system (Addy et al., 2012a). Thus, virulence and pathogenicity factors such as the synthesis of exopolysaccharide and the swimming motility increased in the φRSS1-infected bacterial cells, resulting in early wilting of the host (Addy et al., 2012a). However, filamentous bacteriophages can affect the bacterial wilt pathogen differently. Tomato plants injected with *R. solanacearum* cells infected with φRSM3, another filamentous *Inovirus* closely related to filamentous phage φRSM1 (Yamada et al., 2007; Askora et al., 2009), showed no wilting symptoms (Addy et al., 2012b) (**Table 1**). By contrast, restoration of wilting levels of the pathogen was observed in plants inoculated with bacterial cells infected with the mutant phage φRSM3-ΔORF15. It was then suggested a possible role of the *orf15* of φRSM3 on the repression of *phcA* gene, resulting in loss of virulence (Addy et al., 2012b). Therefore, filamentous bacteriophages can negatively or positively affect the virulence of *R. solanacearum* according to the presence or absence of a repressor gene in the bacteriophage genome (Addy et al., 2012a,b; Yamada, 2013). In fact, there would be at least two groups of filamentous bacteriophages that differentially affect host cell physiology, including virulence regulatory pathways, presumably by mechanisms that might be similar to those affecting other species of phytopathogenic bacteria infected by their filamentous bacteriophages (Askora and Yamada, 2015).

Subsequently, the characterization of 14 bacteriophages isolated from soil in Thailand, belonging to the *Podoviridae* and *Myoviridae* families, revealed that the combination of two podoviruses (J2 and φRSB2) efficiently lysed *R. solanacearum* cells in contaminated soil but, only J2 treatment prevented disease development in tomato plants (Bhunchoth et al., 2015) (**Table 1**). The application of mixtures of other six lytic bacteriophages, isolated from soil in Sri Lanka, to the rhizosphere of tomato plants as a soil drench reduced the incidence of bacterial wilt about 10–20%, either by applying bacteriophage mixtures immediately before inoculation with the pathogen, or watering the soil several times with such mixtures (Kalpage and De Costa, 2014) (**Table 1**).

At present, two bacteriophages (φRSL1 and PE204) proved to be successful for bacterial wilt biocontrol, as it was observed in assays performed in pots with infected tomato plants treated each time with just one of them (**Table 1**). However, host range for these phages only included Asian strains of the pathogen, probably belonging to the present *R. pseudosolanacearum* or *R. syzygii* subsp. *indonesiensis* species (**Figure 1**).

## Patent Literature On Bacteriophage-Based Bacterial Wilt Biocontrol

Three patents have been published in relation to bacterial wilt biocontrol, the last of them with international extension in the United States and China. Japanese patent with publication number JP2005278513 describes three types of *R. solanacearum* bacteriophages with lytic activity against the pathogen, which were isolated from soil in Japan. The phages were initially characterized by their genome size and host range, which was assayed against six Japanese strains isolated from tobacco plants, all of them of race 1: M4S, Ps29, and Ps65 of biovar 3, and C319, Ps72, and Ps74 of biovar 4 (Bhunchoth et al., 2015, Supplementary Table S1) and, therefore, all of them belonging to phylotypes I or IV of the former species complex, and presently not *R. solanacearum* (**Figure 1**). Biocontrol assays were performed against strain C319 by spraying a phage of type 1 or 2 onto tobacco plants or as a soil conditioner (Yamada and Yamamoto, 2005). A second Japanese patent, with publication number JP2007252351 (also published as JP4862154-B2), included a new type of bacteriophage, named φRSA1, with a wider host range with respect to the previous ones. However, although this bacteriophage showed *in vitro* lytic activity against 15 bacterial strains, the bacterial wilt biocontrol efficacy *in planta* was not evaluated (Fujie et al., 2007). In a third Japanese patent, with publication number WO/2012/147928, an agent and a method for preventing bacterial wilt disease were developed, consisting of the application of any of the strains M4S, Ps29, Ps65, and Ps74 infected with φRSM1-type filamentous phage or any of the strains C319, Ps72, and Ps74 infected with φRSM3-type filamentous phage, by injection into the plant stems at 10^5-8^ cells/g plant. This patent was focused on the prevention of bacterial wilt, since the plants injected with bacterial cells containing the filamentous phages integrated in their genomes showed increased resistance to the pathogen for at least 2 months (Yamada et al., 2012). In spite of the publication of these patents, until now no bacteriophage-based product is commercially available to fight bacterial wilt in the field.

## Future Perspective

Further studies on bacteriophage-based bacterial wilt biocontrol methods should focus on biocontrol efficiency under field conditions, a range of susceptible growing crops, and the use of bacteriophage mixtures to prevent the appearance of resistant strains of *R. solanacearum*. Phage bioproduction and formulations to be used as biopesticides for a sustainable and environmentally friendly agriculture would be also necessary. A promising approach to increase the efficacy against the bacterial wilt disease would be the incorporation of phages as biocontrol agents that could be combined with other biocontrol strategies in an integrated control program.

## Author Contributions

BA and EGB wrote the manuscript.

## Conflict of Interest Statement

The authors declare that the research was conducted in the absence of any commercial or financial relationships that could be construed as a potential conflict of interest.
